# Effective COVID-19 Control: A Comparative Analysis of the Stringency and Timeliness of Government Responses in Asia

**DOI:** 10.3390/ijerph18168686

**Published:** 2021-08-17

**Authors:** Shu Chen, Lei Guo, Taghred Alghaith, Di Dong, Mohammed Alluhidan, Mariam M. Hamza, Christopher H. Herbst, Xinqi Zhang, Gabrielle Charis Alano Tagtag, Yi Zhang, Nahar Alazemi, Rana Saber, Reem Alsukait, Shenglan Tang

**Affiliations:** 1 ARC Centre of Excellence in Population Ageing Research (CEPAR), University of New South Wales, Sydney 2052, Australia; 2 School of Risk and Actuarial Studies, University of New South Wales, Sydney 2052, Australia; 3 Duke Global Health Institute, Duke University, Durham, NC 27708, USA; lei.guo@duke.edu (L.G.); xinqi.zhang208@duke.edu (X.Z.); shenglan.tang@duke.edu (S.T.); 4 General Directorate for National Health Economics and Policy, Saudi Health Council, Riyadh 13315, Saudi Arabia; t.alghaith@shc.gov.sa (T.A.); m.alluhidan@shc.gov.sa (M.A.); nalazemi@shc.gov.sa (N.A.); r.saber@shc.gov.sa (R.S.); 5 Health, Nutrition and Population Global Practice, The World Bank, Washington, DC 20433, USA; ddong1@worldbank.org (D.D.); mhamza@worldbank.org (M.M.H.); cherbst@worldbank.org (C.H.H.); yzhang35@worldbank.org (Y.Z.); reemalsukait@gmail.com (R.A.); 6 Division of Health Research, Lancaster University, Bailrigg LA1 4YX, UK; 7 Yale-NUS College, National University of Singapore, Singapore 138527, Singapore; charis.tagtag@u.yale-nus.edu.sg

**Keywords:** COVID-19 control, government response, Asia, stringency, timeliness

## Abstract

Aim: Many governments in East and Southeast Asia responded promptly and effectively at the onset of the COVID-19 pandemic. Synthesizing and analyzing these responses is vital for disease control evidence-based policymaking. Methods: An extensive review of COVID-19 control measures was conducted in selected Asian countries and subregions, including Mainland China, Hong Kong, Taiwan, South Korea, Singapore, Japan, and Vietnam from 1 January to 30 May 2020. Control measures were categorized into administrative, public health, and health system measures. To evaluate the stringency and timeliness of responses, we developed two indices: the Initial Response Index (IRI) and the Modified Stringency Index (MSI), which builds on the Oxford COVID-19 Government Response Tracker (OxCGRT). Results: Comprehensive administrative, public health, and health system control measures were implemented at the onset of the outbreak. Despite variations in package components, the stringency of control measures across the study sites increased with the acceleration of the outbreak, with public health control measures implemented the most stringently. Variations in daily average MSI scores are observed, with Mainland China scoring the highest (74.2), followed by Singapore (67.4), Vietnam (66.8), Hong Kong (66.2), South Korea (62.3), Taiwan (52.1), and Japan (50.3). Variations in IRI scores depicting timeliness were higher: Hong Kong, Taiwan, Vietnam, and Singapore acted faster (IRI > 50.0), while Japan (42.4) and Mainland China (4.2) followed. Conclusions: Timely setting of stringency of the control measures, especially public health measures, at dynamically high levels is key to optimally controlling outbreaks.

## 1. Introduction

Since the onset of the COVID-19 pandemic in 2019 up to 1 August 2021, more than 198.3 million confirmed cases and 4.2 million deaths have been reported globally [1]. Governments have been responding with unprecedented policy responses, aiming to control the spread and mitigate consequences as much as possible. Upon discovering the virus, Mainland China had the highest number of cases and fatalities. However, starting in March 2020, the global epicenter started shifting from Mainland China to Europe and North America. Shortly afterward, in May 2020, significant declines in new cases were observed in several countries; these can be attributed to effective control measures.

Despite more control, new waves of infections have been observed as countries started reducing the stringency of measures and reopening their economies. These waves of infections have been much worse, especially in European countries and the United States since November 2020 [2], which may be the result of lower outdoor temperatures, more indoor activities, holiday gatherings, and the mutations of the coronavirus that have been discovered. In November 2020, a new variant of COVID-19 was discovered in South Africa [3], which requires even more stringent measures because of its more infectious nature. As of July 2021, four major variants are being discovered globally, among which the B.1.617.2 (Delta) has been driving a new surge of COVID-19 cases worldwide [4]. Meanwhile, reduced effectiveness of current vaccines has been found against the widespread Delta variant [5], which highlights the importance of evidence-based effective control measures.

Significant differences across regions in new cases and case fatality rates are observed at the early phases of the pandemic. The daily new confirmed cases per million people were 349.3, 277.8, and 20.7 in Europe, North America, and Asia, respectively, on 15 November, and the case fatality rate was 1.8% in Asia compared with 2.3% and 2.9% in Europe and North America [2]. These differences can be attributed to the variations in government responses. Therefore, it is important to examine common control measures taken by Asian countries in response to the pandemic and to analyze the difference in implementation that may have produced different effects.

Since the outbreak of the COVID-19 pandemic, countries have been adopting various approaches in combating the pandemic based on their different political, socio-economic, cultural, and health system contexts. This has resulted in variations in the ability to effectively control new cases and mortality attributed to COVID-19. Overall, Asian countries and subregions, most of which had prior experience in combating epidemics of novel infectious diseases such as SARS, MERS, and H1N1, have outperformed their counterparts in Europe and North America in controlling the COVID-19 pandemic with timely and effective government responses.

Many studies have been conducted to summarize the control measures implemented globally, including those in Asian countries [6,7,8,9], though few have systematically and comprehensively summarized and analyzed these measures. The Oxford COVID-19 Government Response Tracker (OxCGRT) [10] was developed to evaluate the implementation of the control measures, specifically focusing on variation in the stringency of government response. The most up-to-date OxCGRT contains 19 indicators covering dimensions of containment and closure, economic, health system, and miscellaneous policies [11]. Though informative and widely used, the OxCGRT fails to comprehensively capture several key control measures—for example, establishing coordinated governance has not been included in the OxCGRT. Governance, leadership, and macro-management of the pandemic at the national level is likely to be critically important to the success of its effective control, and therefore should be accounted for when considering the effectiveness of government responses. Furthermore, the OxCGRT evaluates only stringency but ignores timeliness, another essential factor associated with effective control.

This paper aims to examine and compare the timeliness and stringency of control measures implemented during the early phase of the COVID-19 pandemic in selected Asian countries and subregions. The paper focuses on Asian countries and subregions with quality data availability and epidemic control experiences and practices. This was done by extensively reviewing high-quality literature available from 1 January to 30 May 2020. Control measures upon collection were grouped into three categories: administrative, public health, and health system measures. We develop two indices to evaluate and compare the stringency and timeliness of countries’ COVID-19 responses: the Modified Stringency Index (MSI), adapted from the OxCGRT, and the Initial Response Index (IRI). We then propose policy recommendations for future pandemics preparedness.

## 2. Methods

### 2.1. Country/Subregion Selection

China (including Mainland China, Hong Kong SAR/China (hereafter referred to as Hong Kong), and Taiwan/China (hereafter referred to as Taiwan)), South Korea, Singapore, Japan, and Vietnam (hereafter all referred to as the “study sites”), were purposively selected in this review because of the generally good or unique practices manifested in response to the COVID-19 pandemic; their previous epidemic control experiences with SARS, MERS and H1N1; and the availability of comprehensive and comparable data.

### 2.2. Data

Data on the control strategies, policies, and measures taken by the governments of the study sites between 1 January and 30 May 2020 when most of the COVID-19 cases were reported before the current new wave, were collected from various sources. While peer-reviewed publications were prioritized, reliance on gray literature was inevitable because of the limited number of publications available as a result of the short time period. PubMed was first searched using the following key search terms: “COVID-19” (AND), “response” (OR) “experience” (OR) “intervention” (OR) “policy” (AND), the name of specific geographic locations. Gray literature, including articles from reputable magazines and media reports that presented reliable and robustly vetted information, were also utilized. In addition, we collected data from preprints on MedRxiv and unpublished internal reports, which were attained through our collaborations with international and national organizations working in Asia.

Data on the stringency of measures were collected from the University of Oxford for existing indicators. Data on novel indicators that are not part of the OxCGRT were collected from the review results. In addition to policy measures, epidemiological data were also collected to measure the effectiveness of control. These data were collected from authoritative sources including the European Centre for Disease Prevention and Control (ECDC), which has synthesized data from government websites of countries; and the Center for Systems Sciences and Engineering (CSSE) at Johns Hopkins University (JHU) if data on ECDC were not available.

### 2.3. Indicators and Dimensions to Assess COVID-19 Responses

To measure and evaluate governments’ responses, we created key indicators based on the review results. To create the indicators, we first developed an analytical framework to summarize the control measures used across the study sites, grouped into (i) administrative, (ii) public health, and (iii) health system measures. The analytical framework included important indicators, including the location, intervention, implementation date, specific practices, implementation enablers, and barriers.

We then evaluated these key indicators within each category based on the review result and compared them with those included in the OxCGRT. In cases where an indicator was included to measure the same intervention as that in the OxCGRT, the indicator was kept as-is for consistency. If the indicator was included in our framework but was not already part of the OxCGRT, it was added accordingly.

The final set of indicators includes 11 administrative indicators (A), five public health indicators (P), and two health system indicators (H) (Table 1). Seven out of the 18 indicators are novel and are not part of the OxCGRT. These include coordinated governance (A1), legislation and regulations (A2), transparent communications (A11), quarantine (P3), mask-wearing (P5), increasing the supply of personal protective equipment (PPE) (H1), and building/strengthening health facilities (H2).

### 2.4. Development of the MSI and the IRI

To evaluate the stringency of the control measures, we used the scaling methodology of OxCGRT [11] to develop the MSI (see Appendix A Table A1 for the codebook). This consisted of assigning an ordinal value (0, 1, 2, 3, 4) to each indicator based on its stringency level. If an indicator contained a range of information, the ordinal value for measures with a targeted population (instead of the general public) would be deducted by 0.5 to reflect its limited effects. A rescaled score between 0 and 100 was then created based on the ordinal value of each indicator, with missing values given a score of 0. The rescaling process was based on its proportion to the maximum ordinal value of this indicator (see Appendix A). These scores were then averaged among all indicators to get the composite index, MSI. To examine heterogeneity across control measure categories, separate scores for the sub-MSI of administrative and public health indicators were calculated. We did not calculate the sub-MSI score of health system measures as the category consists of only two indicators.

To evaluate and compare the timeliness of responses at the early stage of the pandemic, which is crucial for infectious disease control, we developed the Initial Response Index (IRI). The IRI incorporates timeliness into the stringency index through weighting. Each indicator’s stringency index was assigned a time-based weight, which was calculated using 100 confirmed COVID-19 cases [12] as the threshold and the weight of $Day_{N}$ calculated as:

(1) $$Weight\left(Day_{N}\right)=\{\begin{array}{rr} \frac{\left(100-C_{N-1}\right)}{100}, & C_{N-1}<100\\ 0, & C_{N-1}\geq 100 \end{array}$$

where $Day_{N}$ is the day a control measure was implemented and $C_{N-1}$ is the number of confirmed cases for the day before $Day_{N}$. We took only positive values, and the weighted stringency index of each indicator was averaged to get the IRI value.

### 2.5. Data Analysis

The developed analytical framework was used to examine and compare control measures across the study sites. In addition, we compared and analyzed the change of MSI and the sub-MSI values using average daily MSI scores, and how they changed relative to the disease outbreak by comparing daily new cases. The IRI was also calculated and compared to evaluate variations in governments’ initial responses.

## 3. Results

### Summary of the Government Response among the Study Sites

This analysis highlighted how the study sites have actively been implementing a package of control measures since the onset of the outbreak, covering administrative, public health, and health system dimensions (Table 2). Administrative measures included effective leadership, legislation, and communication in addition to restricting people’s mobility. One common leadership measure taken across most of the study sites is the establishment of coordinated governance. This entailed setting up a national committee or task force consisting of leaders from different ministries/sectors with high-level political leadership at the early stage of the outbreak [13]. Taiwan and Singapore are particularly noteworthy as they commenced these administrative measure–related efforts and achieved this coordination prior to having any confirmed cases [14,15]. With regard to legislation, the key strategies implemented are either to strengthen or issue new legislation and regulations as well as to provide strong enforcement [16,17,18,19]. Governments of the study sites also emphasized transparent information sharing with the public on the epidemic situation [20,21,22,23,24,25]. The key administrative interventions to restrict or limit people’s mobility included border control, lockdown [26,27], and social distancing. To achieve better implementation of lockdown and social distancing policies, governments have taken actions to control traffic, close non-essential businesses, encourage people to stay at home and work from home, and suspend in-person schooling [28,29].

Key public health interventions taken by governments of the study sites concern contact tracing, testing, quarantine, mask-wearing, disinfecting public places, widespread temperature checks, and health education or awareness campaigns. Governments of all the study sites analyzed have taken measures to implement a package of public health interventions, though the components and stringency levels vary. Several similarities in implementation techniques were observed. Most governments within the study sites actively utilized digital and smart tools to achieve more effective implementation with variation [41,42,43,44]. For example, in Japan, a less intrusive method of monitoring was adopted to protect personal privacy. Digital tools have been used for six main purposes: regular documentation and analysis, real-time tracking and alerting, contact tracing, online health consultations and diagnosis, non-human-contact delivery and management, and peer and community reporting (Table 3).

Finally, the last category of health system strengthening included measures taken to facilitate the system with the needed capacity to ensure that it continues functioning. Actions have been taken targeting health service delivery, human resources, health financing, and information systems [45,62,69,70,71]. Despite measures being taken, some systems still suffered significantly, especially at the early stage of the outbreak. With no vaccines or known treatment protocols at the time, governments of the study sites focused on increasing testing capacity for fast and accurate diagnosis, building new health facilities or strengthening the capacity of existing health facilities, increasing the supply of PPE, and improving patient triage to facilitate health service delivery. Actively mobilizing human resources has been key to overcoming shortages of health personnel in response to the outbreak. Active mobilization has been implemented in Mainland China, Singapore, and South Korea [45]. The increase of health funds to relieve the financial burden of seeking care was fundamental to be able to expand testing and treatment coverage. Effective health financing strategies have been implemented by governments of the study sites to cover nearly all costs related to testing and treatment. Strengthening health information systems is also observed as a common measure to ensure data quality. This was especially critical for countries with less-developed systems prior to the outbreak.

**Figure 1:**
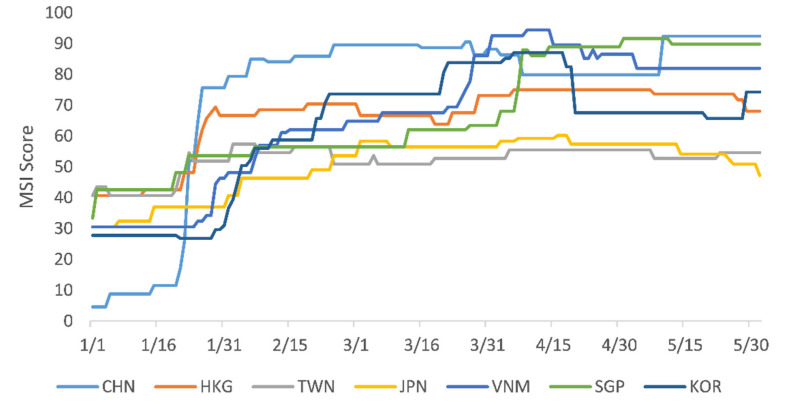
Change in the MSI score in the study sites, 1 January to 30 May 2020. CHN = China, HKG = Hong Kong; JPN = Japan; KOR = South Korea; SGP = Singapore; TWN = Taiwan; VNM = Vietnam. Data sources: ECDC (https://www.ecdc.europa.eu/en/COVID-19/situation-updates (accessed on 1 July 2020)), CSSE at JHU (https://github.com/CSSEGISandData/COVID-19/blob/master/README.md (accessed on 1 July 2020)).

While variations existed among the study sites, generally they have all kept a high stringency level and adjusted it over time (Figure 1). From 1 January to 30 May 2020, the daily average MSI score was 62.8 among the Asian countries. It was highest in Mainland China (74.2), followed by Singapore (67.4), Vietnam (66.8), Hong Kong (66.2), South Korea (62.3), Taiwan (52.1), and Japan (50.3). Observing variations over time, four clear phases with distinct characteristics are evident. The first period is from 1 January to 23 January 2020, when Mainland China fell far behind others—especially Singapore and Hong Kong, which had MSI scores above 40. The second time period is from 23 January to mid-March and is characterized by Mainland China significantly increasing its stringency level to become the highest among the study sites (with mean MSI scores above 85) and others gradually increasing their stringency levels. The third period runs from mid-March to 15 April, when Vietnam, South Korea, and Singapore dramatically increased stringency level while others’ MSI scores remained steady. Finally, the period from 15 April to 30 May can be characterized by reopening, where some countries started to ease stringency levels for economic reasons.

**Figure 2:**
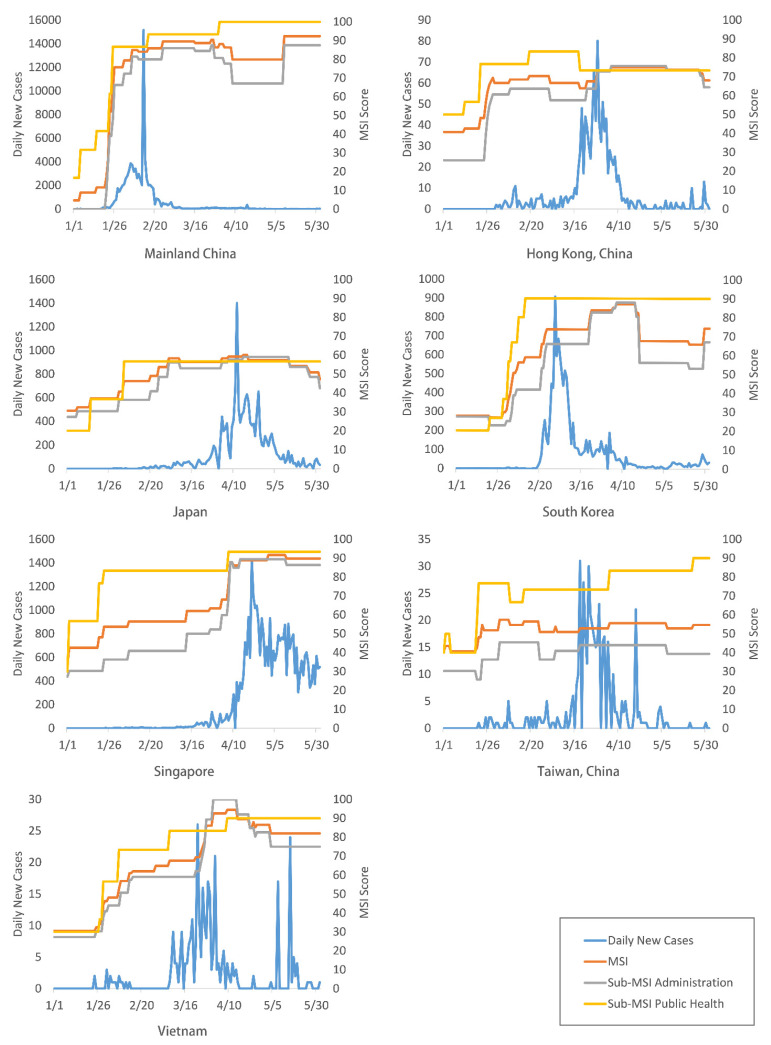
Change of the MSI and sub-MSI relative to disease outbreak in the study sites, 1 January to 30 May 2020. Data sources: ECDC (https://www.ecdc.europa.eu/en/COVID-19/situation-updates (accessed on 1 July 2020)), SSE at JHU (https://github.com/CSSEGISandData/COVID-19/blob/master/README.md (accessed on 1 July 2020)).

Comparing the change in the MSI and the sub-MSI scores with the number of new daily COVID-19 cases (Figure 2), a common trend can be observed: governments increased stringency levels with the acceleration of the outbreak, and stringency was usually highest at the peak outbreak period. Another common feature across the study sites was the central dependency on public health measures. When increasing stringency levels, public health measures were usually prioritized with higher stringency levels than that of administrative measures. On the other hand, when decreasing stringency levels with outbreak decelerations, governments focused on reducing the stringency of administrative measures only, while keeping or even increasing the stringency levels of public health measures.

Results of the IRI show that there are significant variations among the study sites in terms of the timeliness of initial responses. Hong Kong and Taiwan, which are closest to Mainland China and have learned lessons from the SARS outbreak, acted significantly more expeditiously. IRI scores vary, with Hong Kong (62.4), Taiwan (55.9), Vietnam (53.9), and Singapore (50.1) being the highest, while Japan (42.4), and Mainland China (4.2) follow behind.

## 4. Discussion 

Incident and fatality rates of COVID-19 are generally much lower in the study sites than in other areas at the early phases of the pandemic. Through an extensive review, we find that governments of the study sites have actively undertaken a series of administrative, public health, and health system–related measures to control the COVID-19 pandemic.

Though variations in control measures exist, the main measures are consistent across the study sites, providing lessons for disease control. The MSI scores showed variation in the stringency of the implemented control measures across the study sites and within each site over time. The common trends are they usually increased the stringency with the acceleration of the outbreak and prioritized the public health measures. This can be attributed to the flexibility and ease of adaption of public health measures compared to other measures. Results of the IRI provide new insights on the importance of the timeliness of government response and how expeditiously the governments of the study sites acted at the onset of the outbreak. Variations in the timeliness of the initial response were observed: Hong Kong, Taiwan, Vietnam, and Singapore acted much more expeditiously at the onset of the outbreak to implement control measures. Results emphasize the importance of early and timely catchup of implementing control measures, emphasized by the case of Mainland China, which fell behind initially but was able to catch up and surpass others in the study sites.

To our knowledge, this is the first study that has systematically examined control measures by category within the study sites and quantified the timeliness of the initial response. These findings and distilled experiences become especially important to governments globally at the current timing, considering the wide-spread transmission of the Delta variant globally and the reduced effectiveness of the vaccines against it [4,5]. Due to the early identification of COVID-19 cases and overall good performance of the governments of the study sites in responding to the pandemic, previous studies have focused on summarizing the control measures and evaluating their effectiveness, usually done in a single Asian country or subregion such as Mainland China [6,7,9,86], Taiwan [49,87], Singapore [8,88], or South Korea [77,82,89]. Additionally, the OxCGRT, which included 19 indicators to evaluate and compare the stringency level of the control measures across countries, has been used in a few studies to analyze the variation of government response [90] and link the stringency of the OxGGRT with the change of epidemiological indicators [91,92,93].

A distinctive feature of the governments of the study sites is their experience dealing with previous epidemics, thus being prepared with a stock of evidence-based policies. Their experience gave these sites the advantage and ability to react in a timely and effective manner to control the COVID-19 pandemic. Mainland China [94], Hong Kong [95], Taiwan [49,96], and Singapore [8] were severely hit by SARS in 2003. Japan [97] and Taiwan [49] experienced the outbreak of H1N1 in 2009, and South Korea [98] was hit by MERS in 2015. Based on their previous experiences, governments of the study sites took five key actions to improve their response before the COVID-19 pandemic: (i) develop new or amend existing legislation on infectious disease prevention and control [24,94,95,99,100,101]; (ii) set up a government-led organizational structure and mechanism to fight against the emerging infectious diseases [8,74,94,96]; (iii) strengthen infectious disease diagnosis and treatment capacity and reporting system [8,49,94,102]; (iv) improve communication to ensure effective, rapid, and transparent communication between the government and the public [49,94,102]; and (v) strengthen social norms toward controlling infectious diseases among the general population, such as voluntary mask-wearing among the general public.

Based on the results discussed above, we propose several key policy recommendations for COVID-19 pandemic control and pandemic preparedness. First, establishing a well-functioning legislative and organizational structure to guide the response is vital. Second, timely activation of the response mechanism and undertaking evidence-based control measures promptly and dynamically is critical. Public health control measures are particularly important and should febe prioritized and not eased, even with outbreak deceleration. Third, timely and transparent communications with the public and other key stakeholders are highly imperative to reduce panic and boost people’s trust in government. Fourth, ensuring universal access to testing and treatment for all people is critical. Fifth, strengthening the health system is an on-going process and has to be prioritized for effective emergency preparedness response. Last, strengthening surveillance and reporting systems of infectious diseases cannot be ignored, especially in countries where information systems are less developed. In addition, all central governments should develop a realistic and practical roadmap for reopening their economy and businesses.

The current study has several limitations. Firstly, this study is not representative, since a purposive selection of sites was conducted. Additionally, the information collected is not balanced across the study sites, largely because of language barriers and data availability. For example, we find Japan has published significant amounts of COVID-19-related information in Japanese, and evidence related to Vietnam is less rich. Also, though the evaluation indices add indicators to previously used indices, they contain only 18 indicators. These have not captured all the important control measures found based on the review, especially in the health system group, because of limited data availability. We also rely on the number of cases to assess the timeliness of the response in the IRI; this is potentially biased, as the number of confirmed cases is highly dependent on the testing capacity. However, these are the best data available. In addition, the 100-case threshold used for early response is arbitrary and based on literature, given that there is no universal standard. Finally, the data was collected from January to May 2020, failing to include the recent response changes due to the COVID-19 variants (e.g., Delta variant). However, the control measures do not change much except for that some governments tightened them up, such as prolonging the quarantine days, expanding the definition on close contacts et al. We believe lessons drawn from the current study apply to the control of the Delta variant and future pandemics. Future studies can improve on the methodology as more data and information become available and can expand the range of countries to explore how the MSI and IRI correlate with the change of COVID-19 epidemiological indicators to provide feasible policy implications.

## 5. Conclusions

Governments of the study sites in Asia have implemented a series of similar administrative, public health, and health system measures to control the COVID-19 pandemic, though with variations in components, stringency, and timeliness of the initial response. Results based on extensive literature review and in-depth analysis reveal that setting and adapting the stringency of control measures, especially public health measures, according to outbreak accelerations, and reacting expeditiously are key to controlling outbreaks. Such results imply that governments can enhance their response to better control COVID-19 through strengthening its legislative and organizational structure, timely activating the response mechanism and undertaking evidence-based control measures, establishing timely and transparent communications with the public and other key stakeholders, ensuring universal access to testing and treatment for all, and improving the surveillance system.

## Figures and Tables

**Table 1:** List of the indicators included in the evaluation/measurement indices.

Indicator ID	Included in OxCGRT	Indicator Name	Description
Administrative			
A1	No	Coordinated governance	Recorded establishing a national task force/committee consisting of leaders from different ministries/sectors
A2	No	Legislation and regulations	Recorded developing or amending existing legislation and regulations for COVID-19 control
A3	Yes	Border control	Recorded border control for COVID-19 control
A4	Yes	Canceling public events	Recorded canceling public events
A5	Yes	Restrictions on gatherings	Recorded the cut-off size for bans on private gatherings
A6	Yes	School closures	Recorded school and university closures
A7	Yes	Workplace closures	Recorded workplace closures
A8	Yes	Stay-at-home requirements	Recorded “shelter-in-place” and otherwise confine to the home
A9	Yes	Closing public transportation	Recorded public transportation closures
A10	Yes	Restrictions on internal movement	Recorded restrictions on moving between regions/cities
A11	No	Transparent communications	Recorded establishing transparent communications working mechanism
Public health			
P1	Yes	Contact tracing	Recorded government policy on contact tracing after a positive diagnosis
P2	Yes	Testing policy	Recorded government policy on who has access to testing
P3	No	Quarantine	Recorded government policy on quarantine
P4	Yes	Public information campaigns	Recorded presence of public information campaigns on COVID-19
P5	No	Mask-wearing	Recorded mask-wearing policy for COVID-19 control
Health system			
H1	No	Increasing the supply of PPE	Recorded actions on increasing the supply of PPE for health professionals
H2	No	Building/strengthening health facilities	Recorded actions to build or strengthen health facilities

PPE = Personal Protective Equipment.

**Table 2:** Summary of government responses to COVID-19 in the study sites, 1 January to 30 May 2020.

Key Control Measures	Geographic Coverage	Common Practice	Variation in Implementation
**Administrative**			
Establishing coordinated governance [13,14,15,30,31,32,33,34]	All but JP	Established at the very early stage of the outbreak, with high-level political leadership and commitment, and multi-sectoral or multi-ministerial coordination.	Governments differ in the timeliness of establishing this coordinated high-level leadership (e.g., HK, TW, SG, and VN did so even before there were ≤5 confirmed cases)
Amending/adding legislations or regulations [14,16,17,18,19,35,36]	CHN, TW, SG, SK and VN	Governments usually add new regulations or pass new acts related to COVID-19 control, and impose severe penalties to violators for enforcement.	New rules differ in legislative nature (Act, Regulations, etc.), regulated thematic areas and enforcement stringency (e.g., SG and SK have issued strict penalty rules for violation).
Transparently sharing epidemiological status [20,22,23,24,25,32]	All	Press conferences were held across the study sites and media tools were used to present timely updates to the public.	Governments differ in the timeliness of establishing these transparent sharing mechanisms. Variations also exist in format and frequency.
Implementing mobility restrictions interventions [26,27,28,29,37,38,39,40]	All	Governments have used their executive power to implement administrative interventions to restrict people’s mobility, including border control, lockdowns, and social distancing. Lockdowns and social distancing were achieved through interventions such as traffic control, non-essential business closures, shelter-in-place policies, in-person school closures, etc.	Study sites differ in the package of interventions implemented and the stringency of their implementation.
**Public Health**			
Aggressive contact tracing aided by digital tools [41,42,43,44]	All but JP	Contact tracing is done through massive and careful epidemiological investigations among close contacts of people who test positive. Given its labor-intensive nature, most countries mobilized human resources and adopted digital tools to assist with the process.	All are similar except for Japan, which uses a different cluster-based approach to trace contacts and did not implement aggressive measures, to protect personal privacy.
Extensive testing [45,46,47,48,49,50,51,52]	All but JP	Governments focused on conducting nucleic acid amplification tests to detect the virus in suspected cases and in close contacts of confirmed cases.	All are similar except for Japan, which had adopted a restrictive testing approach aiming to not overwhelm its healthcare system.
Strict quarantine [48,53,54,55,56,57,58,59,60,61]	All but JP	Quarantine has been implemented among people with infection risk, including close contacts, suspected cases, travelers, and recovered COVID-19 patients.	All are similar except for Japan, in which quarantine policies are comparatively loose and target only travelers.
Mask-wearing (compulsory and voluntary) [43,62,63,64,65,66,67]	All	Both healthy and infected people are encouraged to wear masks in public places.	Compulsory mask-wearing was observed in some study sites from the very beginning (e.g., CHN, VN), while voluntary mask-wearing was observed in others (HK, SK and JP). Some adjusted their policy to make mask-wearing compulsory for all, including for healthy people (SG and TW).
Disinfecting public places [68]	All	Regular and thorough disinfection of public places, especially those with large population mobility and density.	No variations
Widespread temperature screening	All	Wide thermal equipment was set up in public places such as subway entries and airports and manual temperature checks were given to people before entering a residential area or closed building	No variations
Health education/awareness campaigns [45]	All	Health education and awareness campaigns were usually implemented on TV, social media, and phones and in public places, etc.	No variations
**Health system**			
Improving health service delivery [45,62,69,70,71]	All	Governments focused on increasing testing capacity, building new health facilities, increasing the supply of PPE, and improving the triage of patients to improve health service delivery.	Governments differ in the timeliness of initiating these actions, and in the resources utilized for implementation.
Mobilizing the health workforce [45]	CHN, SG, and SK	Human resources for health were mobilized from other regions to support the most heavily affected places.	Study sites differ in the scale of health workforce mobilization and the types of health workers mobilized.
Increasing health financing [24]	All	Effective financing strategies were implemented to cover the majority, if not all, of the testing and treatment costs through health insurance plus special subsidies.	Study sites differ in the costs and population coverage (eg. SK had everything covered).
Enhancing health information systems [45]	CHN	Careful monitoring, review, and timely publication of cases and a strong oversight and accountability mechanism	Data unavailable

CHN = Mainland China; JP = Japan; SG = Singapore; SK = South Korea; TW = Taiwan; VN = Vietnam.

**Table 3:** Summary of key digital and smart tools used for controlling COVID-19 in the study sites, 1 January to 30 May 2020.

Purpose	Data Collected	Intrusiveness	Examples
Regular documentation and analysis	Health, travel history, drug purchase behaviors, etc.	High	CHN, TW, HK—border control health declaration by scanning a QR code [72] CHN—“Health code” [73], drug purchase direct reporting system [34], health self-reporting system, online registration system for employees to contain risk, etc. TW [74]—big data system to integrate health insurance and customs and immigration database; a digital platform to operate a nationalized system of mask distribution, and the “NHI Express App” for users to check mask availability SK—big data system to collect people’s credit card transaction data, CCTV footage, and mobile phone locations etc.
Real-time tracking and alert system	Health, travel history, GPS location, CCTV footage, etc.	High	TW [14]—electronic security monitoring system and SMS notifications; social distancing alert app HK [75]—wristbands and mobile app SK [24]—cell phones vibrate with emergency alerts when cases are nearby
Contact tracing	Health, basic socio-economic status, travel history, credit card, CCTV footage, etc.	High	CHN [76], TW [14], SK [77], VN [78]—using big data to trace contacts SG [42]—TraceTogether (mobile app) VN [41]—Bluezone (mobile app)
Online health consultation and diagnosis	Health, travel history, and other relevant information per request	Medium	CHN [79]—remote consultation with doctors online; AI-assisted diagnosis JP [80], VN [81]—remote consultation SK [82]—self-diagnosis app
Non-human-contact delivery and management	N/A	Low	CHN [79,83]— Drones: delivery of goods and medical samples; disinfection; crowd management Robots: meals delivery and disinfection
Peer and community support	N/A	Low	CHN [84]—WeChat group to coordinate needs for living essential goods for collective order and delivery SG [85]—“Stay Home for Singapore” portal

CHN = Mainland China; JP = Japan; SG = Singapore; SK = South Korea; TW = Taiwan; VN = Vietnam; N/A = not available.

## Data Availability

The data used for analysis are all available in the manuscript and appendix.

## Appendix A

**Table A1:** Indicator codebook.

ID	Indicators Name	Meaurment	Coding Instructions
A1	Coordinated governance	Ordinal	0—No measures 1—Health sector coordination 2—Established multi-sector coordinated leading/governance team Blank—No data
A2	Legislation and regulations	Binary	0—No measures 1—Amended existing or developed new legisltions or regulations for COVID-19 Blank—No data
A3	Border control	Ordinal	0—No measures 1—Open to all countries with travelers * screening 2—Border closure with targeted countries 3—Border closure with all countries Blank—No data
A4	Canceling public events	Ordinal scale + binary for geographic scope	0—No measures 1—Recommended cancellation 2—Required cancellation Blank—No data 0—Targeted 1—General Blank—No data
A5	Restrictions on gatherings	Ordinal scale + binary for geographic scope	0—No restrictions 1—Restrictions on very large gatherings (the limit is above 1000 people) 2—Restrictions on gatherings between 101 and 1000 people 3—Restrictions on gatherings between 11 and 100 people 4—Restrictions on gatherings of 10 people or less Blank—No data 0—Targeted 1—General Blank—No data
A6	In-person school closures	Ordinal scale + binary for geographic scope	0—No measures 1—Recommended closing 2—Required closing at some levels 3—Required closing at all levels Blank—No data 0—Targeted 1—General Blank—No data
A7	In-person workplace closures	Ordinal scale + binary for geographic scope	0—No measures 1—Recommended closing (or recommend work from home) 2—Required closing (or work from home) for some sectors or categories of workers 3—Required closing (or work from home) for all-but-essential workplaces (e.g., grocery stores, doctors) Blank—No data 0—Targeted 1—General Blank—No data
A8	Stay-at-home requirements	Ordinal scale + binary for geographic scope	0—No measures 1—Recommended not leaving the house 2—Required not leaving the house with exceptions for daily exercise, grocery shopping, and “essential” trips 3—Required not leaving the house with minimal exceptions (e.g., allowed to leave once a week, or only one person can leave at a time, etc.) Blank- No data 0—Targeted 1—General Blank—No data
A9	Closing public transportation	Ordinal scale + binary for geographic scope	0—No measures 1—Recommended closing (or significantly reduced volume/route/means of transport available) 2—Required closing (or prohibited most citizens from using it) Blank—No data 0—Targeted 1—General Blank—No data
A10	Restrictions on internal movement	Ordinal	0—no measures 1—Recommended not to travel between regions/cities 2—Internal movement restrictions in place Blank—Bo data 0—Targeted 1—General Blank—No data
A11	Transparent communications	Ordinal	0—No measures 1—Establishing a transparent communications mechanism by the government though with pauses 2—Establishing nonstop transparent communications by the government on COVID-19 Blank—No data
P1	Contact tracing	Ordinal	0—No contact tracing 1—Limited contact tracing; not done for all cases 2—Comprehensive contact tracing; done for all identified cases Blank—No data
P2T	Testing policy	Ordinal	0—No testing policy 1—Only those who both (a) have symptoms AND (b) meet specific criteria (e.g., key workers, admitted to hospital, came into contact with a known case, returned from overseas) 2—Testing of anyone showing COVID-19 symptoms 3—Open public testing (e.g., “drive through” testing available to asymptomatic people) Blank—No data
P3	Quarantine	Ordinal	0—No quarantine policy 1—Only targeted to international travelers 2—Quarantining all persons with infection risks, including travelers, close contacts, suspected cases and recovered patients Blank—No data
P4	Public information campaigns	Ordinal	0—No COVID-19 public information campaign 1—Public officials urging caution about COVID-19 2—Coordinated public information campaigns (e.g., across traditional and social media) Blank—No data
P5	Mask-wearing	Ordinal	0—No measures 1—Recommended mask-wearing in public places 2—Compulsory mask-wearing in public places Blank—No data
H1	Increasing the supply of PPE	Ordinal	0—No measures 1—Some action to increase the supply of PPE 2—Used all-societal resources to the increase supply of PPE Blank—No data
H2	Building/strengthening health facilities	Ordinal	0—No measures 1—Renovated health facilities or strengthen infection control in health facilities 2—Built new health facilities for COVID-19 patients only Blank—No data

* Different from OxCGRT’s coding: code 1 (Screening) and 2 (Quarantine arrivals from high-risk regions) in the OxCGRT were merged into code 1 in our codebook.
